# Access to menstrual hygiene products through incentivised, community-based, peer-led sexual and reproductive health services before and during the COVID-19 pandemic: findings from the Yathu Yathu trial

**DOI:** 10.1186/s12889-022-12915-5

**Published:** 2022-03-21

**Authors:** Hensen B, Gondwe M, Phiri M, Schaap A, Simuyaba M, Floyd S, Mwenge L, Sigande L, Shanaube K, Simwinga M, Fidler S, Hayes R, Ayles H

**Affiliations:** 1 Clinical Research Department, London School of Hygiene and Tropical Medicine, Keppel Street, London, WC1E 7HT UK; 2 Zambart, Lusaka, Zambia; 3 Department of Infectious Disease Epidemiology, London School of Hygiene and Tropical Medicine, London, UK; 4 Imperial College and Imperial College NIHR BRC, London, UK

## Abstract

**Background:**

Access to affordable and effective menstrual hygiene products (MHP) is critical to the menstrual health of adolescent girls and young women (AGYW). In this mixed-methods analysis, we use data from a programme delivering comprehensive sexual and reproductive health (SRH) services to describe access to MHP and how COVID-19-related closures affected access to MHP; we use qualitative data to understand AGYW’s experience accessing products during the study.

**Methods:**

Between September 2019-January 2021, we used data routinely collected from ten Yathu Yathu hubs offering community-based, peer-led SRH services to adolescents and young people aged 15–24. Hubs offered free MHP (primarily disposable pads) as a service. To incentivise service access, a “loyalty” card system was embedded within Yathu Yathu, allowing individuals to gain points for services accessed and redeem rewards using these points. Branded pads, tampons and reusable pads were among available rewards. We describe access to service pads and to reward MHP, and use logistic regression to investigate factors associated with accessing pads and reward products before (Sept 2019-March 2020) and after (July 2020-Jan 2021) COVID-19-related closures. Focus group discussions explored accessibility of offering MHP through hubs.

**Results:**

Between September 2019-January 2021, 6374 AGYW made 34,116 hub visits to access an SRH service and/or redeem a reward. At 30% of these visits, AGYW accessed any MHP. Before COVID19-closures, an average of 17% of monthly visits were for freely-available disposable pads compared to 34% after hubs reopened (*p* < 0.001). Results were similar for reward visits. Overall, 63% of 6374 AGYW collected pads as a service at least once. Prior to COVID19-closures, AGYW aged 18–24 were more likely to access service pads than adolescents (15–17-years). After reopening, access was lower among older AGYW. Prior to hub closures, uptake of reward MHP was higher among AGYW with some secondary education but not after reopening. Discussions revealed that, for adolescents aged 15–19, COVID-19-related hub closures required reverting to using ineffective materials to manage menstruation.

**Conclusion:**

Availability of MHP through Yathu Yathu provided a large number of AGYW with access to these products. Hubs seemed particularly important for adolescent girls. Community-based, peer-led hubs should be considered as spaces to provide AGYW access to affordable and effective MHP.

## Introduction

Menstruation remains a social taboo and many adolescent girls and young women (AGYW) have limited information about menstruation and access to the menstrual hygiene products (MHP) [1, 2]. Compounded by gender inequalities, poverty, and poor access to water and sanitation facilities, particularly in low- and middle-income countries, many AGYW face an unmet need for menstrual health, defined as “a state of complete physical, mental, and social well-being and not merely the absence of disease or infirmity, in relation to the menstrual cycle” [3]. This unmet need has implications for AGYW’s participation in education, and in social and economic activities [2, 4, 5].

In support of universal menstrual health, UNICEF has outlined a framework for menstrual health and hygiene programming consisting of four pillars: 1) social support; 2) knowledge and skills; 3) facilities and services, and 4) a choice of appropriate and affordable MHP [1]. Globally, the cost of MHP is a critical barrier to AGYW’s access to MHP; as such, the term “period poverty” was coined to advocate for universal access to menstrual hygiene products. In low- and middle-income countries, including Zambia, studies show that competing priorities, including food and school fees, means money for MHP is often unavailable [6]. In 2020, with the outbreak of COVID-19, AGYW access to MHP was likely further constrained by measures to control the spread of the SARS-CoV-2 virus [7]. Understanding the implications of ongoing COVID-19 control measures for access to MHP is important to identify and implement strategies to maintain AGYW access to MHP during the pandemic and future disease outbreaks requiring similar control efforts.

In response to broader unmet need for sexual and reproductive health (SRH) services among adolescents and young people aged 15–24 (AYP) in Lusaka, Zambia, Yathu Yathu was implemented in September 2019 to provide community-based, peer-led and free SRH services to AYP through ten spaces (hubs) in two urban communities. Among the services available at the hubs are MHP, including (reusable) sanitary pads, tampons and menstrual cups, and comprehensive sexuality education, which includes sessions on menstruation and menstrual hygiene. Through these services, Yathu Yathu aims to address two of four pillars for menstrual health and hygiene programming: knowledge and skills on how to use different MHPs and provision of an affordable range of MHP. Yathu Yathu also aims to provide support for use of MHP through the peer support workers.

Using data routinely collected on use of services at the hubs, we describe attendance to and uptake of MHP at Yathu Yathu hubs. In mid-March 2020, Zambia reported its first cases of COVID-19. Subsequently, the government implemented restrictions on public gatherings, closed all schools, and mandated physical distancing and use of masks in public spaces [8]. Although COVID-19 cases rose slowly in subsequent months, evidence suggests national and international control measures had an almost immediate adverse effect on household incomes in Lusaka [9]. In response to the growing number of COVID19 cases, the study team decided to close the hubs for three months between April-July 2020. We hypothesised that these closures, compounded by COVID-19 control measures in place nationally, may have negatively affected AGYW’s access to MHP, and resulted in higher uptake of products after reopening for fear that hubs might close again. As such, we compared uptake before and after hub closures, explored whether characteristics of the AGYW accessing products differed before and after the COVID-19 related hub closures, and use qualitative data to understand AGYW experiences in accessing products during the study period.

## Methods

### Study location and population

The Yathu Yathu intervention is being implemented in 10 geographical areas within two peri-urban communities in Lusaka, Zambia. Each area has a population of ~ 2,350 individuals aged 15–24 years. Prior to Yathu Yathu implementation, all households in the 10 geographical areas were enumerated and all AYP offered a prevention points card (PPC), a “loyalty” card that allows AYP to gain points for services accessed and use these points to redeem health-related rewards. AYP were informed of the availability of SRH services through the Yathu Yathu hubs and that, using their PPC, they could gain points and redeem rewards for accessing services. The impact of Yathu Yathu on knowledge of HIV status is being evaluated in an ongoing cluster randomised trial (CRT) [10].

### Yathu Yathu

Yathu Yathu was co-designed with AYP using qualitative research and discrete choice experiments [11]. The intervention consists of the delivery of comprehensive SRH services, including HIV testing, condoms and other contraceptives, through community-based hubs. MHP, primarily disposable sanitary pads but also reusable cups, are available as a service. Six-hundred cups were donated to Yathu Yathu from the company *Chicashana*. Services are delivered by peer support workers, who manage the day-to-day activities at the hub, and are supported by a nurse and supervisor.

In addition to service provision, access is incentivised by the PPC system. The rewards available through this system include soap, higher-quality, branded disposable sanitary pads and tampons, reusable pads and deodorant. The number of points earned per service accessed was discussed with AYP during co-development and is dependent on the psychological difficulty of accessing services. The points required per reward are dependent on cost of the rewards (1-point equivalent to 0.05 Kwacha (USD 0.002)). Some rewards could be accessed as a bundle, for example, a razor and pack of disposable pads. All AYP are able to access services, even without a PPC, but cannot collect points without a PPC.

Between September 2019 and January 2020, Yathu Yathu was implemented in a pilot phase. During this phase, evidence on acceptability and reach, among other implementation domains, were used to adapt the intervention in support of increased coverage. During this phase, AGYW could earn 65 points for collecting disposable sanitary pads. Branded tampons were redeemable as a reward using 560 points, branded pads for 380 points, reusable sanitary pads for 3000 and the razor and pads bundle for 513 points.

After the pilot phase, the PPC system was adapted such that the collection of free pads no longer allowed AGYW to earn points and the “cost” of reward MHP was reduced to below market value (140 points each; USD 0.33; reusable pads reduced to 1500 points and razor/pads bundle to 297 points). These changes were implemented on February 18^th^, 2020. Subsequently, on April 1^st^ 2020, hubs were closed in response to the growing number of COVID-19 cases in Zambia. Hubs reopened on July 1^st^ 2020, with adaptations made to limit the number of AYP attending the hub at one time, mandate mask wearing and handwashing prior to entering the hubs, and implement an appointment system.

### Data sources

For the quantitative analysis, we used data routinely collected from the PPC between September 2, 2019 and January 23, 2021. This system collects data on the date of a visit to the hub, what services were accessed during a visit to a hub, and the date rewards were redeemed at a hub and what reward was redeemed. We also used data collected during the enumeration, including age, sex, educational attainment and marital status of the AYP enumerated in the study communities.

A narrative qualitative research approach was [12] used to explore the acceptability of offering MHP through Yathu Yathu hubs and used the “follow the thread” approach [13] to understand AGYW’s experiences of accessing MHP during the study period, particularly during COVID-19 related hub closures. Between December 11–22, 2020, two focus group discussions (FGD) were conducted with adolescent girls (aged 15–19) and young women (aged 20–24) who had accessed different MHP from the hubs (*n* = 17). Based on this inclusion criteria, a simple random list of AGYW who had accessed MHP, stratified by age group [15–19 years and 20–24 years), was generated. This list of participants was given to the hub staff, who either phoned or physically followed up the AGYW to invite them to participate in the qualitative research. The hub staff also approached AGYW as they were accessing services to invite them to participate in the FGDs. Throughout the qualitative data collection, efforts were made to ensure credibility of findings. At the design stage, FGDs were considered the most appropriate data collection method to understand acceptability among AGYW and experiences accessing MHP throughout the study period. The data collection guides were developed by the social science team with input from the broader study team to ensure that the tools would capture these domains. A female research assistant (MG), familiar with the study communities, facilitated the FGDs in a language preferred by the participants at the health facility or a conveniently located place within the community. To ensure accurate presentation of the data, transcripts were checked against the FGD recordings and were read several times to derive themes representative of the domains of interest.

### Outcomes and explanatory variables of interest

The quantitative analysis includes two outcomes: uptake of service MHP and use of PPC points to redeem a reward MHP, both outcomes were estimated before and after COVID-19-related hub closures. At the time of this study, menstrual cups were a relatively new product option for AGYW in the study communities and only 600 were stocked through the donation. As such, we describe the number of cups collected, but do not include cups in subsequent analyses. For the second outcome, the variable was coded 1 if AGYW collected any MHP as a reward, including the razor/pads bundle, and 0 if they collected a reward but not MHP as a reward. Factors explored for their association with these outcomes included: age, educational attainment and marital status, all reported during enumeration, and, for uptake after closures, whether AGYW attended the hub for a service or reward before COVID-19 related closures and collected service or reward MHP, respectively.

### Data analysis

Analyses were restricted to AGYW attending a hub at least once. For services and rewards, we first described the total number of visits to the hubs for services and/or rewards. We described the percentage of these visits where AGYW collected service or reward MHP, respectively. Using the percentage of service or reward MHP collected per month, we measured the average percentage of visits in the pilot phase (Sept 2 2019 – Feb 17 2020), post-pilot phase (Feb 18 2020 to March 31 2020), and the period after (Jun 2020- Jan 2021) hub closures. By hub, we then compared the average in the period before COVID-19-related closures (Sept 2019 – Mar 2020) to after and the average in the post-pilot phase (Feb 18 2020 to March 31 2020) to after, using the paired t-test.

Subsequently, we described uptake of service MHP among all AGYW attending the hubs, and explored individual-level factors associated with uptake of service pads at both time points (before and after COVID-19-related closures). Finally, we estimated uptake of reward MHP among all AGYW attending hubs to redeem a reward and explored individual-level factors associated with this outcome at both time points. For our risk factor analyses, we used logistic regression, with fixed effect to account for clustering by the ten zones.

FGDs were audio recorded and field notes were taken during data collection. The audio recordings were transcribed verbatim and field notes written as a summary report. MG read the transcripts to become familiar with the data and identify key emerging issues. The emerging issues were categorised into themes using matrix tables. Themes were identified from the key topic areas, including: acceptability of hubs for accessing MHP, impact of COVID-19-related closures on access to MHP and experiences of accessing MHP after the re-opening of the hubs. Thick descriptions of the summary data including participants’ quotes were included in the matrices and were used to interpret the data and made up the key study findings.

### Ethical considerations

The University of Zambia Biomedical Research Ethics Committee (007–04-19) and the London School of Hygiene and Tropical Medicine (17104) approved the Yathu Yathu study. AYP aged 18 to 24 provided written informed consent before PPC distribution. For adolescents aged 15–17, parents/guardians provided written informed consent and adolescents written informed assent.

## Results

**Fig. 1:**
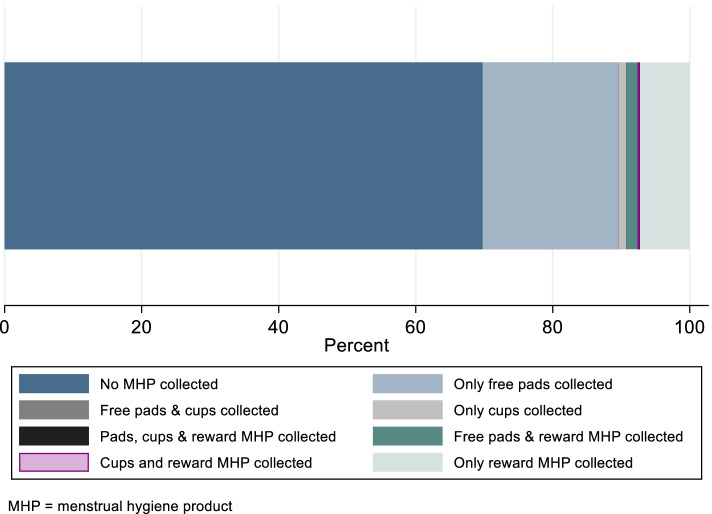
Uptake of disposable pads and reusable cups as a service, and of menstrual hygiene products as a reward per visit to the Yathu Yathu hubs, September 2019 – January 2021

Between September 2, 2019, and January 23, 2021, 6374 AGYW made 34,116 visits to the hubs to access an SRH service and/or redeem a reward. At almost one-third (30.2%, *n* = 10,306) of these visits, AGYW accessed pads, a cup and/or a reward MHP (Table 1 & Fig. 1). During these 10,306 visits, 11,217 MHP were distributed; the majority being freely-available reusable pads (66.0%; 7405). Of the 600 menstrual hygiene cups, 535 (89.2%) were distributed.

**Table 1:** Number of service and reward visits by AGYW aged 15–24 and collection of pads and reward MHP at Yathu Yathu hubs during the different phases of the study period (September 2020 – January 2021)

Overall number of visits for a **service and/or reward**	34,116				
Overall number of visits where any MHP collected	10,306				
Overall % of visits where MHP collected	30.2				
	**Pilot Phase**	**Post-adaptation Phase**	**Entire phase pre-COVID19-related closures**	**Post-COVID19-related closure phase**	
	**02 September 2019—17 Feb 2020 (5.5 months)**	**18 Feb 2020—31 March 2020 (1.5 months)**	**02 Sept 2019—31 Mar 2020 (7 months)**	**1 July 2020—23 January 2021 (7 months)**	*p*-value*
Number of visits for a **service**	17,201	1773	18,974	13,097	
Number of **service** visits where pads collected	2401	645	3046	4359	
Overall % of **service** visits where pads collected	14.0	36.4	16.1	33.3	
Hub-level average % of visits pads collected	14.7	36.9	16.8	33.5	< 0.001
Number of visits for **rewards**	5314	707	6021	8109	
Number of **reward** visits where MHP collected	451	135	586	2691	
Overall % of **reward** visits where MHP collected	8.5	19.1	9.7	33.2	
Hub-level average % of visits where MHP collected	8.8	20.4	10.2	33.5	< 0.001

*MHP* Menstrual hygiene product, *AGYW* Adolescent girls and young women

^*^Paired t-test, comparing pad/MHP collection before and after COVID-19 related closures across hubs

During this period, 32,071 visits were made to access SRH services (Table 1). Before hub closures, an average of 16.8% of visits were for pads compared to 33.5% after hubs reopened (*p* < 0.001). In the two months after the pilot phase, the proportion of visits for a pad increased significantly relative to the pilot phase (36.9%, *p* < 0.001) but there was no difference between this period and after hubs reopened (36.9% vs 33.5%, respectively; *p* = 0.22). Overall, 5383 AGYW made 14,130 visits to the hubs to redeem rewards. Before hubs closures, an average of 10.2% of reward visits were for MHP compared to 33.5% after hubs reopened (*p* < 0.001). Between February–March 2020, after implementing the adaptations, there was evidence for increased collection of MHP relative to the pilot phase (20.4%; *p* = 0.01) and evidence that collection was lower in this period compared to when hubs reopened (20.4% vs 33.5%, respectively; *p <* 0.001).

### Individual-level factors associated with accessing menstrual hygiene products at Yathu Yathu hubs

**Fig. 2:**
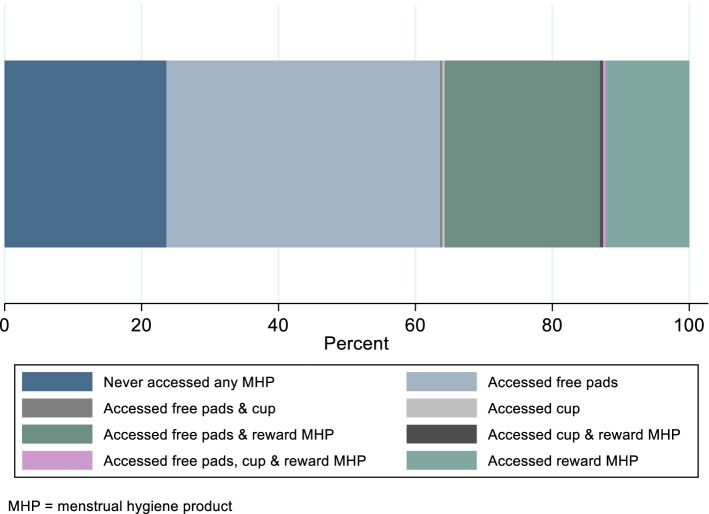
Uptake of disposable pads and reusable cups as a service, and of menstrual hygiene products as a reward by individual adolescent girls and young women aged 15-24, at any time during the study period September 2019 – January 2021

Among the 6374 AGYW attending the hubs, 40.1% (*n* = 2555) were aged 20–24 and 78.3% (*n* = 4991) were never married. Overall, 4872/6374 (76.4%) accessed pads or cups as a service and/or MHP as a reward (Fig. 2).

Overall, 63.4% (*n* = 4038/6374) of AGYW collected freely-available pads at least once. Prior to COVID-19-related closures, older AGYW aged 18–24 (64.4%, *n* = 575/893) were more likely to access pads than adolescents aged 15–17 (57.9%, *n* = 1005/1736; adjOR: 1.34 95%CI 1.14, 1.59). After reopening, there was no change in uptake among adolescents aged 15–17 but uptake was lower among older AGYW (Table 2). In both time periods, AGYW who were married were less likely to access pads. Collecting pads prior to COVID-19-related hub closures was strongly associated with accessing pads after hubs reopened (adjOR = 1.37 95%CI: 1.18, 1.59; Table 2).

**Table 2:** Characteristics of adolescent girls and young women aged 15–24 visiting the Yathu Yathu hubs and accessing pads before and after COVID19-related hub closures, September 2019-January 2021

	**All AGYW visiting hubs**	**Characteristics of AGYW visiting hubs & accessing pads before hub closures**				
	**Number (column %) of all AGYW visiting the hubs**	**Number of AGYW (column %)**	**Number (row %) of AGYW collecting pads**	**Crude OR**	**Adjusted OR + **	***p*-value**
**Overall**	6374	3910	2344 (60.0)			
**Age group**						
15–17	2385 (37.4)	1736 (44.4)	1005 (57.9)	1.0	1.0	0.003
18–19	1434 (22.5)	893 (22.8)	575 (64.4)	1.34 (1.13, 1.59)	1.34 (1.13, 1.59)	
20–24	2555 (40.1)	1281 (32.8)	764 (59.6)	1.07 (0.92, 1.25)	1.07 (0.92, 1.25)	
**Educational attainment**^a^						
None or some/complete primary	1531 (24.0)	999 (25.6)	552 (55.3)	1.0	1.0	0.09
Some/completed secondary	4843 (76.0)	2911 (74.5)	1792 (61.6)	1.21 (1.04, 1.41)	1.14 (0.98, 1.33)	
**Marital**^b^						
Single/Never married	4991 (78.3)	3268 (83.6)	1997 (61.1)	1.0	1.0	< 0.001
Married/Cohabiting	1383 (21.7)	642 (16.4)	347 (54.1)	0.75 (0.63, 0.90)	0.68 (0.56, 0.84)	
**Characteristics of AGYW visiting hubs & accessing pads after the hub closures**						
	**Number of AGYW (column %)**	**Number (row %) of AGYW collecting pads**	**Crude OR**	**Adjusted OR + **	***p*-value**	
**Overall**	**5695**	**2783 (48.9)**				
**Age group**						
15–17	2153 (37.8)	1218 (56.6)	1.0	1.0	< 0.001	
18–19	1281 (22.5)	609 (47.5)	0.68 (0.59, 0.78)	0.68 (0.59, 0.78)		
20–24	2261 (39.7)	956 (42.3)	0.55 (0.49, 0.62)	0.55 (0.49, 0.62)		
**Educational attainment**^a^						
None or some/complete primary	1338 (23.5)	665 (49.7)	1.0	1.0	0.15	
Some/completed secondary	4357 (76.5)	2118 (48.6)	1.00 (0.88, 1.13)	1.10 (0.97, 1.25)		
**Marital**						
Single/Never married	4477 (78.6)	2300 (51.4)	1.0	1.0	< 0.001	
Married/Cohabiting	1218 (21.4)	483 (39.7)	0.61 (0.53, 0.69)	0.77 (0.66, 0.89)		
**Visited hub before COVID-19 related closures**						
No	2464 (43.3)	1174 (47.7)	1.0	1.0	0.08	
Yes	3231 (56.7)	1609 (49.8)	1.05 (0.95, 1.18)	0.90 (0.81, 1.01)		
**Collected pads during visit before COVID-19 related closures (*N* = 3231)**^a^						
No	1150 (35.6)	520 (45.2)	1.0	1.0	< 0.001	
Yes	2081 (64.4)	1089 (52.3)	1.33 (1.15, 1.54)	1.37 (1.18, 1.59)		

*AGYW* Adolescent girls and young women aged 15–24, *OR* Odds ratio, *95%CI* 95% Confidence interval

All models account for clustering by enumeration zone

+ All adjusted for age group

^a^Further adjusted for marital status

^b^Further adjusted for educational attainment

Before hub closures, 17.1% (*n* = 464) of 2713 AGYW who redeemed a reward opted for MHP. After reopening, 47.3% of 4252 AGYW who redeemed a reward collected MHP. Prior to hub closures, redeeming reward MHP was higher among older AGYW and associated with having completed secondary education or higher (18.7% vs up to complete primary: 12.3%; adjOR = 1.53 95%CI: 1.17, 1.99). After closures, there was no evidence for an association with age or educational attainment. As with freely-available pads, married AGYW were less likely to redeem reward MHP throughout the study period, and, among AGYW who obtained a reward prior to closures, collecting reward MHP before closures was strongly associated with accessing reward MHP after hubs reopened (Table 3).

**Table 3:** Characteristics of adolescent girls and young women aged 15-24 visiting the Yathu Yathu hubs for a reward and collected reward pads before and after COVID19-related hub closures, September 2019-January 2021

AGYW visiting hubs for a reward and pads collected as rewards before hub closures					
	Number (column %) of AGYW visiting hubs for a reward	Number (row %) of AGYW collecting reward pads	Crude OR	Adjusted OR	*p*-value
Overall	2713	464 (17.1)			
**Age group**					
15-17	1258 (46.4)	205 (16.3)	1.0	1.0	0.03
18-19	621 (22.9)	129 (20.8)	1.35 (1.06, 1.73)	1.35 (1.06, 1.73)	
20-24	834 (30.7)	130 (15.6)	0.97 (0.76, 1.24)	0.97 (0.76, 1.24)	
**Educational attainment**^a^					
None or some/complete primary	676 (24.9)	83 (12.3)	1.0	1.0	0.002
Some/completed secondary	2037 (75.1)	381 (18.7)	1.59 (1.22, 2.05)	1.53 (1.17, 2.00)	
**Marital**^b^					
Single/Never married	2313 (85.3)	418 (18.1)	1.0	1.0	0.004
Married/Cohabiting	400 (14.7)	46 (11.5)	0.60 (0.43, 0.83)	0.58 (0.40, 0.84)	
**AGYW visiting hubs for a reward and pads collected as rewards after hubclosures**					
	**Number (column %) of AGYW visiting hubs for a reward**	**Number (row %) of AGYW collecting reward pads**	**Crude OR**	**Adjusted OR**	***p*-value**
**Overall**	4252	2010 (47.3)			
**Age group**					
15-17	1619 (38.1)	782 (48.3)	1.0	1.0	0.27
18-19	956 (22.5)	468 (49.0)	1.01 (0.86, 1.19)	1.01 (0.86, 1.19)	
20-24	1677 (39.4)	760 (45.3)	0.91 (0.79, 1.04)	0.91 (0.79, 1.04)	
**Educational attainment**^a^					
None or some/complete primary	996 (23.4)	440 (44.2)	1.0	1.0	0.28
Some/completed secondary	3256 (76.6)	1570 (48.2)	1.10 (0.95, 1.27)	1.09 (0.94, 1.26)	
**Marital**					
Single/Never married	3345 (78.7)	1641 (49.1)	1.0	1.0	<0.001
Married/Cohabiting	907 (21.3)	369 (40.7)	0.71 (0.61, 0.83)	0.69 (0.58, 0.83)	
**Visited hub for rewards before COVID-19 related closures**					
No	2670 (62.8)	1247 (46.7)	1.0	1.0	0.18
Yes	1582 (37.2)	763 (48.2)	1.14 (1.00, 1.30)	1.10 (0.96, 1.26)	
**Collected MHP during reward visit before COVID-19 related closures (*N*=1582)**^a^					
No	1247 (78.8)	565 (45.3)	1.0	1.0	<0.001
Yes	335 (21.2)	198 (59.1)	1.68 (1.31, 2.17)	1.63 (1.27, 2.11)	

*AGYW* Adolescent girls and young women aged 15-24,* OR* Odds ratio, *95%CI* 95% Confidence interval

All models account for clustering by enumeration zone, with adjusted models all adjusted for age

^a^Further adjusted for marital status

^b^Further adjusted for educational attainment

#### AGYW’s experience of accessing menstrual hygiene products during the study period

In FGDs, hubs were considered private spaces, where AGYW could be free and open, with products offered alongside instructions on use. AGYW described how the hubs made them feel more comfortable to access MHP, in contrast to the experience of accessing pads from shops.

*“R6: … they are very much suitable because at those hubs where we go that is where they teach us how to do it, how to wear them, they give us more knowledge on these things” (FGD, 15–19)*

*“R2: … for me there were times when going to buy the pads from a shop, I would be feeling shy like that. I would feel ashamed because maybe they are thinking that I am on my periods. But this side, I do not know maybe because the peers, the Yathu Yathu crew, they are young, our age-mates like that … then you feel free like that, they won’t even question you, they know that yeah you are going to use this for that. But in a shop, it was difficult for me.” (FGD 20-24 Community (C)1)*

AGYW recounted how, in the absence of money, they would use pieces of cloth and cotton during menstruation. As such, hub closures in response to COVID-19 negatively affected AGYW, particularly adolescents aged 15–19. During closures, AGYW resorted to using the materials used prior to the availability of free pads through Yathu Yathu. One AGYW mentioned that she was “disturbed” and found herself having to choose between buying snacks or pads.

*“R3: We suffered. When you think, that Yathu Yathu has been closed, what will I do? And then parents would ask us, ‘There is nothing that I can do, just wear the piece of cloth’.” (FGD, 15–19 C2)*

*“R4: It’s a situation whereby Yathu Yathu has made you get used to wearing pads, then you think, I go back to pieces of clothes, mmh I start getting burnt. It became difficult for me. I would get disturbed whereby, I would be forced, when I have a K1 (Kwacha), failing to even eat properly, to buy [name of snack], the way you would usually eat, you will keep it, so that you can save and buy pads” (FGD, 15-19 C2)*

The thought of having to revert back to alternative materials was stressful, as they can cause burning, smelling, itching and, for some, fear of staining left them socially isolated.

*“R1: Yes, we went to pieces of clothes but they are not stable, you would fold it, it is thick yes but when blood starts to come out, it is not like a pad which absorbs, but the pieces of material stains. If you are attending (on periods) then there is no going anywhere” (FGD, 15-19 C2)*

Once hubs reopened, AGYW continued to access services; however, fears that the hubs would close again led to some AGYW choosing to hoard pads.

*“R3: …But when we heard that Yathu Yathu was opened, we went, we would get two (2 packs), because in case you close we would have already gotten two or when it finishes we would still have.” (FGD, 15-19 C2)*

## Discussion

Our analysis shows that there is a need for improved access to MHP for AGYW. With approximately 5000 AGYW attending community-based, peer-led hubs offering SRH services between September 2019 and January 2021 to collect MHP, one option that appears acceptable and accessible is to deliver MHP through community-based hubs. Despite removing points redeemable for collection of free disposable pads, the number and percentage of AGYW accessing pads as a service increased with time. With changes to the points required to redeem reward MHP and after COVID-19-related closures, the number of AGYW attending the hubs to access rewards increased and almost half used their points to obtain MHP. Accessing freely-available pads was associated with age and marital status throughout the study period, and accessing MHP as a reward associated with marital status throughout. Reducing the “cost” of reward MHP likely contributed to increased use of points for MHP, with age and educational attainment no longer associated with accessing reward MHP after hub closures.

Our quantitative analysis shows an increase in access to MHP at the Yathu Yathu hubs over time. For rewards, this increase was significant immediately after changes to the number of points required to “buy” better quality reward MHP. These findings reiterate the importance of providing MHP at reduced prices or free of charge [14]. Since late 2019, period poverty has likely been exacerbated during the COVID-19 pandemic [15]. On 12 January 2021, in response to increasing concerns regarding period poverty, Scotland became the first country to pass a law mandating AGYW’s legal right to free access to sanitary pads and tampons [16]. In Tamil Nadu, India, school closures in response to the epidemic led to a programme to distribute pads via schools being interrupted [15]. Our qualitative findings show that AGYW, who became accustomed to accessing MHP at the hubs, struggled to access MHP during hub closures and experienced anxiety at having to revert to alternative, less hygienic products, which have been shown to be associated with urogenital infections in India [17]. Upon reopening, AGYW reported, and our quantitative findings reflect, “hoarding” pads for fear that hubs might close again. Reducing the cost of MHP is critical to facilitate universal access to a range of MHP, with ongoing supply of free products critical.

Our study suggests that community-based spaces that offer a range of SRH services are acceptable and accessible places for AGYW to access free sanitary pads. In particular, these spaces are important for adolescents aged 15–17, who likely have less agency and financial autonomy for how and where to access MHP. A qualitative study in rural Uganda reported similar findings, with AGYW unable to access MHP due to financial constraints and lack of availability [18]. Our findings that, independent of age, married and/or cohabiting AGYW were less likely to access MHPs at the hubs may reflect greater financial independence to purchase products as a result of partnerships.

Yathu Yathu provides an opportunity to inform adolescents and young people about menstruation. Limited knowledge of menstruation has implications for broader sexual and reproductive health [1], while inadequate access to MHP means many AGYW use ineffective and unhygienic products, risking urogenital infections [17, 19] and absenteeism from school, and other social and economic activities [2, 20–22]. In a cross-sectional survey in Ethiopia, 28% of adolescents aged 10–19 did not have information on menstruation and menstrual hygiene before menarche; 60% had poor menstrual hygiene practices, with only 42% using commercially made pads [23]. Studies have shown that providing AGYW with access to MHP and education can increase school attendance [24]. Providing a space where AGYW can access appropriate and free MHP, feel supported in doing so and receive information on menstruation addresses activities within three of the four pillars outlined in UNICEF’s framework for effective menstrual health and hygiene programming. Ensuring water and sanitation facilities in schools, workplaces and other settings are adequate is required to complement programmes delivering MHP, social support, and information [1, 25].

Yathu Yathu not only provides an opportunity to inform adolescents and young people about menstruation and menstrual hygiene, but to incentivise attendance to these educational sessions through the PPC system. The PPC system allowed AGYW more choice by providing access to additional products at reduced “cost”. By incentivising service access, Yathu Yathu provides an opportunity for AGYW to access a range of MHP, including branded disposable pads, reusable pads, and tampons. Despite increased choice, the majority of AGYW opted to use their points to redeem disposable pads. This preference may be driven by a lack of knowledge of how to use tampons, the high “cost” of reusable relative to disposable pads and/or the inconvenience of reusable products, which require access to water, time to wash and space to dry the pads. A qualitative study in Malawi similarly found a preference for disposable pads among girls aged 10–18 [14]. In a study in Zimbabwe, over 80% of females aged 16–24 attending community-based spaces offering SRH opted to access menstrual hygiene products, with uptake higher among 16–19 year-olds and 80% opting for reusable pads over menstrual hygiene cups [22]. Considering the potential benefits of reusable products, at the environmental- and individual-level, and the need to provide AGYW with choice, increased support is needed for AGYW to use such MHP, through more information and exposure to alternative MHP.

Our study is subject to limitations. In the absence of a control, we cannot assess whether Yathu Yathu increased access to MHP. However, as described, the impact of Yathu Yathu on knowledge of HIV status and other outcomes is being evaluated in an ongoing CRT. We only include data from two FGD, among AGYW who accessed MHP at the hubs. AGYW who did not attend the hubs or access MHP at the hubs may have reported different experiences during hub closures. Despite limitations, this analysis uses data routinely collected by the PPC system during service delivery. At the time of the study, almost 70% the AGYW enumerated in the study communities had attended a hub at least once. As such, the data provides important insights into the health seeking behaviours of AGYW, and how an incentivised system can support access to essential healthcare products. The ongoing CRT will provide rigorous information on whether Yathu Yathu increased AGYW’s access to MHP by comparison of the Yathu Yathu intervention arm to a comparison arm.

## Conclusion

The availability of appropriate and affordable MHP through Yathu Yathu, which delivers activities relevant to three of the four pillars in UNICEF’s framework for effective menstrual health and hygiene programming [1], provided a high number of AGYW with access to these products. More adolescent girls aged 15–17, who likely have less economic independence relative to young women aged 18–24, accessed these products at the hubs, particularly after hub closures. Ensuring adolescent girls are able to access MHP, particularly during COVID-19, is of critical importance to safeguard their participation in education and the workplace and ultimately their broader physical and mental well-being. With adolescents more likely to access products and educational attainment not associated with accessing pads as a service, community-based, peer-led spaces may remove some of the economic barriers to accessing MHP; however, schools and other community-based venues may also prove appropriate places to deliver free MHP. Period poverty remains a global public health issue; increased availability of MHP at low to no cost is essential.

## Data Availability

The datasets analysed for the current analysis are available from the corresponding author on reasonable request. The University of Zambia Biomedical Research Ethics Committee (007–04-19) and the London School of Hygiene and Tropical Medicine (17104) approved the Yathu Yathu study. Adolescents and young people aged 18 to 24 provided written informed consent to participate in the study. For adolescents aged 15–17, parents/guardians provided written informed consent and adolescents written informed assent to participate. For individuals with lower literacy and therefore unable to read or write, a witness could sign the consent form on behalf of the participant. All methods were carried out in accordance with relevant guidelines and regulations.
